# Difficulty-skill balance does not affect engagement and enjoyment: a pre-registered study using artificial intelligence-controlled difficulty

**DOI:** 10.1098/rsos.220274

**Published:** 2023-02-01

**Authors:** Joe Cutting, Sebastian Deterding, Simon Demediuk, Nick Sephton

**Affiliations:** ^1^ Digital Creativity Labs, University of York, York YO10 5DD, UK; ^2^ Dyson School of Design Engineering, Imperial College London, London SW7 2BX, UK

**Keywords:** difficulty, skill, flow, engagement, artificial intelligence

## Abstract

How does the difficulty of a task affect people's enjoyment and engagement? Intrinsic motivation and flow theories posit a ‘goldilocks’ optimum where task difficulty matches performer skill, yet current work is confounded by questionable measurement practices and lacks scalable methods to manipulate objective difficulty-skill ratios. We developed a two-player tactical game test suite with an artificial intelligence (AI)-controlled opponent that uses a variant of the Monte Carlo Tree Search algorithm to precisely manipulate difficulty-skill ratios. A pre-registered study (*n* = 311) showed that our AI produced targeted difficulty-skill ratios without participants noticing the manipulation, yet different ratios had no significant impact on enjoyment or engagement. This indicates that difficulty-skill balance does not always affect engagement and enjoyment, but that games with AI-controlled difficulty provide a useful paradigm for rigorous future work on this issue.

## 1. Introduction

The idea that an inverted-U-shaped curve describes the relation between stimulus and response dates back to the earliest modern psychological models of human behaviour by Yerkes and Dodgson [1] and Wundt and his eponymous Wundt curve [2]. Optimal experience in areas such as interest, taste or music [2–4] has been found in the ‘golden middle’ of this curve, corresponding to a stimulus of moderate magnitude. Across work, education, sports and leisure, the most popularly known inverted-U model comes from flow [5] and related theories of intrinsic motivation [6], which propose that tasks with moderate difficulty relative to a person's skill level are the most motivating, leading to subjective enjoyment and behavioural engagement. Flow theory in particular posits a balance of task difficulty and skill as the most important antecedent of optimal experience or flow in an activity [7]. This model of difficulty-skill balance is widely used in job and task design at work [8,9], instructional design in education [10], coaching and training in sports [11,12], and especially game design for entertainment [13,14] and learning [7]. It is directly implemented in adaptive systems that try to automatically optimize users' experience and performance, including adaptive guidance and learning task selection in intelligent tutors [15], task allocation in crowdsourcing [16,17], and so-called matchmaking [18] and dynamic difficulty adjustment (DDA) systems [19] in games, which aim to present players with balanced opponents and game challenges.

However, despite the widespread adoption of the difficulty-skill balance model, empirical work on the subject is far from uniform—studies have repeatedly found limitations, moderators and data patterns contradicting the model (e.g. [20–22]). More importantly, prior work suffers from significant methodological issues. First, existing studies use a wide variety of measures and calculations for difficulty, skill and difficulty-skill balance as independent variables, which the most recent meta-analysis found simply ‘too heterogeneous to meaningfully aggregate’ [23]. This goes hand in hand with a similar breadth of dependent variable measures for enjoyment and engagement, spanning different scales and subscales of flow, intrinsic motivation or unvalidated scales [23]. Such *measurement flexibility* opens massive researcher degrees of freedom and makes comparing and aggregating results hard [24]. Second, the majority of studies (every single one from 28 studies identified in the cited meta-analysis [23]) measure difficulty, skill and difficulty-skill balance with *self-report scales*, which have come under increasing critique especially in media effects research for their systematic biases and high variance [25]. Third, existing self-report studies operationalize balance either with unvalidated scales—showcasing a ‘measurement schmeasurement’ attitude [26] that threatens construct and overall study validity—or with a subscale of the same flow scale which is then used as the dependent variable, inducing significant spurious correlations [23]. All this ties directly, fourth, into the *theoretical flexibility* [27,28] of the difficulty-skill balance construct: to our knowledge, flow and intrinsic motivation research have yet to produce an unambiguously formalized prediction of what constitutes a ‘balanced’ difficulty-skill ratio. When pressed, flow researchers [22] point to Csikszentmihalyi & Nakamura's [29] proposition that ‘experiences that one believes are in the neighbourhood of a 50/50 balance are experienced as enjoyable’, but what does ‘50/50’ refer to here? Frequency of success versus failure? Perceived odds? The referent of ‘50/50’ is not specified by Csikszentmihalyi, and so researchers fall back on self-report measures that leave it to their participants' private and varied conceptions and feelings to determine what counts as ‘balanced.’ Yet such self-report measures always run the risk of tautology or the ‘jangle fallacy’ [26]: who would logically ever say that a ‘too high’ difficulty is more enjoyable than one that feels ‘balanced’? Similarly, what if we found a data pattern where engagement or enjoyment peaked at 95% winning odds, or at a 2 on a 7-point perceived difficulty scale? Unless we have some agreed formal operationalization of balance, it is difficult to make cumulative progress establishing whether there is a sweet spot ratio that holds across persons and situations, and if so, at which value [28].

Just as importantly, even if we had a well-validated, standardized self-report scale and formal prediction for subjectively *perceived* balance, self-report measures are markedly less useful for practitioners who wish to set and adapt task difficulty to a sweet spot. An objective behavioural or outcome measure of balance is far easier and less intrusive to implement than a system that has to elicit regular self-reports. Consider the fact that while there are plenty of research prototypes of game DDA systems and intelligent tutors that use affect sensing or self-report as inputs [30,31], we know of no single reported commercial implementation using anything but behavioural or outcome measures.

Fifth and finally, most of the few studies that manipulate and measure such objective difficulty [30,32–35] do so by setting and analysing fixed *absolute* difficulty levels at the *cohort* level, rather than *relative* to *individual* skill, that is, difficulty-skill *ratios*, as theory stipulates. Hence, these studies lack basic construct validity. At best, they rely on the untested assumptions that (i) skill is normally distributed across their participant sample, and in such a way that (ii) whatever the study sets as moderate difficulty actually meets the sample's mean skill. Under these assumed conditions and random assignment, fixed cohort-level difficulty manipulation would turn varying player skill into mere additional uncontrolled variance. But any multi-modal or comparatively skewed skill distribution would violate those assumptions and invalidate derived inferences.

To our knowledge, only five studies have directly tested relations between an objective, individual-level skill-difficulty ratio and a fully separate enjoyment or engagement measure, with contradictory results: one study found an inverted-U relation between enjoyment and balance in self-selected online Chess matches [36], operationalizing balance as relative material strength: how many standard pawn unit equivalents a player had more or less on the board than their opponent at the time of measurement. A follow-up study on digital games [37] manipulated difficulty-skill using a ‘Wizard of Oz’ technique in which the experimenter stopped the game and measured engagement at particular score ratios. While it found the same inverted-U pattern, the study suffers from a lack of transparency and precision, as well as from possible experimenter bias. A large-scale online maths game study, in contrast, found a linear relation between success rate and behavioural engagement—the easier the game, the longer people played [38]. A follow-up study [20] could replicate this pattern, but found it became an inverted-U if and only if players consciously self-selected a difficulty setting, instead of difficulty being randomly assigned and not revealed. Finally, a study on teaching children to read [39] operationalized and manipulated difficulty as the proportion of successful trials, but found no difference in engagement between a 60% success rate and an 80% success rate.

In sum, across fields, the inverted-U model of difficulty-skill balance remains widely used despite or because of the lack of robust cumulative evidence—evidence that is hard to come by due to a lack of methods for formally operationalizing and testing objective difficulty-skill ratios at the individual level.

As the few existing studies furnishing the latter show, games offer well-controlled experimental environments [40] for investigating objective difficulty-skill ratios. In competitive games, (artificial) opponent strength gives a ready operationalization of objective difficulty, while measures of objective player skill and skill-difficulty ratios can be derived from player performance in the form of win rates and game scores against given opponents: assuming a fair, skill-based game, the outcomes of (repeated) player-opponent matches directly express the ratio of player strength (= skill) to opponent strength (= difficulty), while match outcomes of varying players against a fixed opponent express varying player skills and vice versa. This approach is already widely used in multi-player ranking or matchmaking systems like Elo [41] for Chess or TrueSkill [42] for digital games, which parse past match data to calculate play strength ratings for players that allow to estimate winning odds for future matches, and thus to determine and make matches with desired ‘balanced’ winning odds.

However, logistical challenges have held back the experimental use of this approach: ranking systems like Elo require large datasets of past matches to accurately assess player strength, which limits the participant pool to those for whom such data exists. Manipulating difficulty by matching player participants with confederate opponents of a desired strength poses even greater logistical challenges. Hence previous studies in this vein have relied on either non-experimental analyses of unmanipulated naturally occurring data [20], or human confederates self-handicapping, which is by necessity imprecise [37].

One principally scalable form of adjusting difficulty-skill ratios are DDA systems as used in commercial games. While there are many different approaches to DDA [19], the majority relies on a function that assesses online player performance with some in-game outcome measure (e.g. position in a race, frequency of failing a level) and then adjusts game parameters (e.g. strength and number of opponents) if player performance deviates from a predetermined optimum. Unfortunately, current commercial DDA systems are usually hard-coded and black-boxed: researchers do not know and cannot control how the DDA manipulates the game state or what it sets as optimal. In the worst case, this leads to tautological study designs that operationalize ‘balance’ as ‘using DDA’ (e.g. [30,32]).

**Figure 1.**
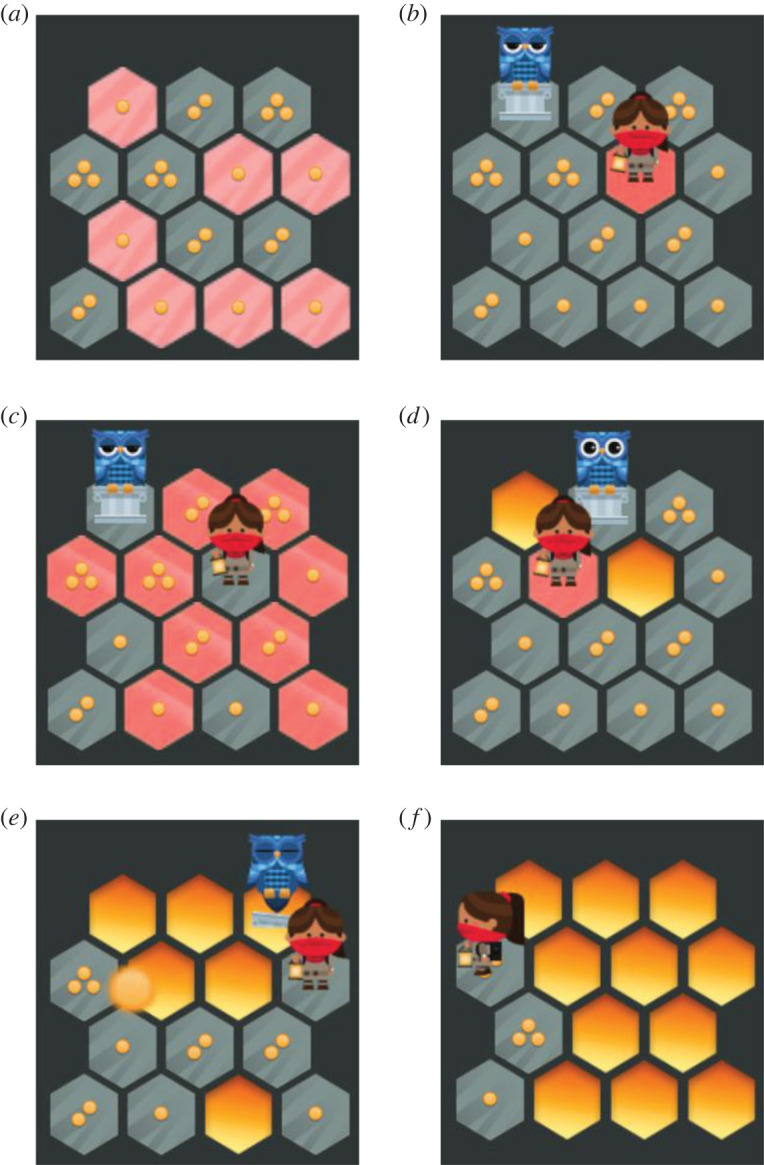
The game *Explorers vs Owls* is a simple turn-based tactical two-player game. Apart from initial tile placement, the game is fully transparent and deterministic, making it as dependent on and expressive of player's tactical skills as *Go* or Chess. (*a*) The game is played on a grid of hexagonal tiles each of which contains 1–3 coins. The winner is the player who collects the most coins. (*b*) The human player controls the explorers, the AI player the owls. Both start the game by placing one figure at a time on any tile which contains one coin. (*c*) Once all figures have been placed, players take turns. On their turn, the player selects one figure and moves it in anywhere in a straight line that is not blocked by another figure or a lava tile. (*d*) When a player moves a piece, the tile they moved from turns to lava and their score increases by the number of coins on the tile they move to. (*e*) If a figure is ever surrounded by lava, the edges of the board, and/or other figures such that it cannot move, the figure is removed from the board. (*f*) The game continues until all figures are removed from the board. The winner is the player with the highest score = collected coins.

To overcome this issue and provide future research with an experimental test suite for rigorous large-scale experimental studies, we created a competitive, skill-based two-player online game, *Explorers* *vs* *Owls* (figure 1), using an artificial intelligence (AI) opponent that allowed us to precisely control difficulty-skill ratios thanks to a DDA technique we created, Monte Carlo Tree Search Outcome Sensitive Action Selection (MCTS OSAS, see *Materials and methods*, figure 3) [43,44]. MCTS OSAS algorithms are based on the MCTS [45–47] algorithm which has proven capable of producing very strong-playing AI opponents even in games like *Go*, where it is difficult to measure a players' performance or the value of a move until the end of the game [48].

We performed a pre-registered (https://osf.io/f4vkm) randomized between-subject study (*n* = 311) to validate our game test suite and test the proposition that difficulty-skill ratios affect engagement and enjoyment. To rigorously test the latter, we pre-registered two main and two connected hypotheses:

**H1:** Experiential enjoyment will significantly differ between game conditions with different difficulty-skill ratios.
**H1a:** If H1 is supported, the game condition with the highest closeness of results will have significantly higher experiential enjoyment than either other condition.
**H2:** Behavioural engagement will significantly differ between game conditions with different difficulty-skill ratios.
**H2a:** If H2 is supported, the game condition with the highest closeness of results will have significantly higher behavioural engagement than either other condition.

Following [37], we operationalized difficulty-skill ratio as *Player Win Margin* (PWM), calculated as the participants’ end-game score minus the opponent AI's score, where a 0 is a draw, a positive PWM a player win and a negative PWM a player loss.

To validate our approach, we compared the target skill-difficulty ratios of three different, randomly assigned conditions (easy, balanced and hard) with their actual end-game scores. Following [29], we specified difficulty-skill balance (the *balanced* condition) as an equal relation of player and AI score, i.e. a target PWM of 0 (draw). We set *easy* (PWM = 15) and *hard* conditions (PWM −15) at 50% of the median player score (30.5) during pilot studies which were supported by player self-reports showing that participants experienced these as markedly different difficulties. We chose a draw as the ‘balanced’ condition as prior work by Csikszentmihalyi and others had operationalized objective balanced challenge similarly [36,37]. If an actual optimum PWM exists, we grant that this may sit at a different point than our balanced condition, or even at a point outside our three conditions. We opted to use three markedly distinct difficulty-skill ratios to, first, validate the effectiveness of the DDA technique, and second, test whether different difficulty-skill ratios have any impact on experience and enjoyment. If both of these conditions are met, then subsequent studies can determine the optimum difficulty-skill ratio for a positive experience. For validating our approach, we pre-registered the following hypotheses:

**H3:** There will be a significant difference in PWMs between conditions.
**H3a:** if H3 is supported, there is a significant difference between each condition in the direction that the AI was aiming for.

Finally, as player awareness of DDA has been found to confound enjoyment as it undermines perceived agency [49], we also measured feelings of agency to check whether our manipulation was noticed.

## 2. Materials and methods

For this experiment, we developed a simple tactical two-player board game called *Explorers* *vs* *Owls* (figure 1), modelled on the popular board game *Hey That's My Fish!* and other positional games such as *Go*. To maximize dependence on isolated tactical skill, the game is fully transparent and deterministic (apart from the initial random board set-up), meaning player and AI opponent can fully determine available moves, counter-moves and their immediate consequences, and it is turn-based, requiring no hand-eye coordination or fast reflexes. We ensured the game remained accessible for our broad participant pool: players can learn the rules and finish a game in a few minutes, while gameplay has enough strategic depth to require considerable tactical skill to play well. Individual game rounds lasted 2–3 min. The tutorial level which teaches the game is shown in figure 1; the follow-on levels are the same except they are played on a larger 7 × 7 grid and each player has three pieces rather than one. An online version of the whole study which does not save data can be viewed at http://joecutting.com/demos/EvOExp1/.

**Figure 2.**
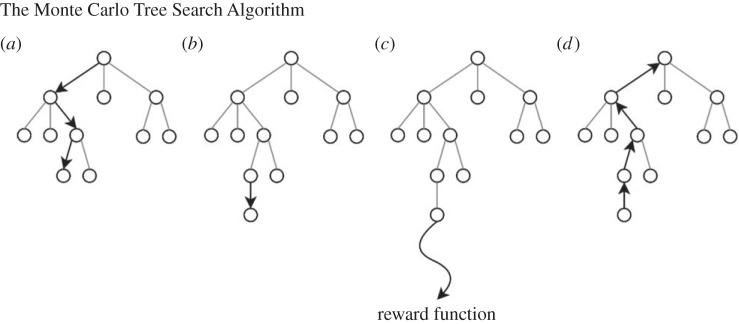
The MCTS algorithm creates a tree of possible game moves and iterates around this tree to select the optimum move. Each iteration has four steps: (*a*) selection: start at the root node and move down the tree until a leaf node is reached, using an ‘urgency’ function to choose which child nodes to explore. (*b*) Expansion: add one random unexplored move node to the leaf node. (*c*) Simulation: choose random moves until the game ends and then calculate the reward function for the final game state. (*d*) Backpropagation: the result of the reward function is used to amend each previously visited node until the root is reached. These values then feed into the ‘urgency’ function used in subsequent iterations during the selection step.

To manipulate difficulty-skill ratios in *Explorers* *vs* *Owls*, we implemented an AI opponent that dynamically adjusted difficulty using an MCTS OSAS algorithm developed by one of our team members [44], which itself is based on the popular MCTS algorithm [45]. Like similar so-called tree search algorithms, MCTS builds up a branching tree of possible game moves and counter-moves to some number of *n* steps into the future, evaluating each game state according to some reward function (figure 2). To efficiently manage this rapidly expanding search space, MCTS selectively and probabilistically subsamples branches which promise better outcomes. This efficiency enables the move tree to be searched many moves ahead, and often to the end of the game. This has proven to create strong opponents even in games like *Go* where it is difficult to measure a players' performance until the end of the game: the MCTS-based *AlphaGo* [48] has beaten some of the top human *Go* players.

**Figure 3.**
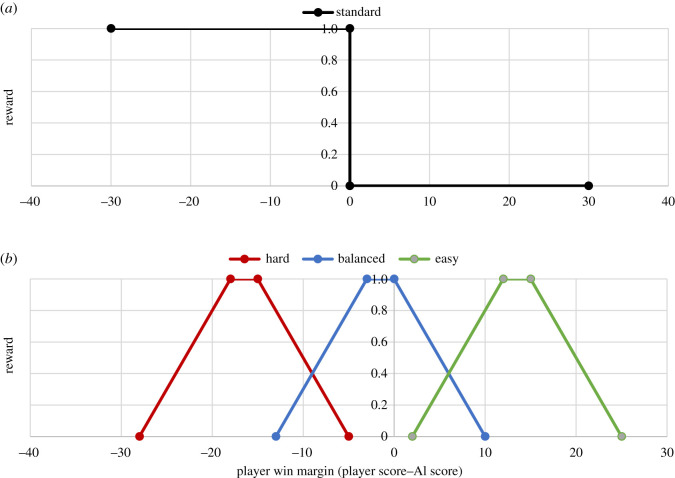
The MCTS algorithm aims for a final game state with the highest reward value. Figure (*a*) shows the reward function for standard MCTS, whereas (*b*) shows the reward functions used by MCTS OSAS (True POSAS variant) for three different game targets (hard, balanced and easy). In MTCS OSAS, the reward value is determined by the target ‘PWM’ (player score – AI score) for each condition. The balanced condition aims for a PWM of around 0 to create a draw, the easy condition aims for the player to win by 15 points and the hard condition aims for the player to lose by 15 points.

Standard MCTS implementations in games are set up to play as strongly as possible—e.g. their reward function might simply return 1 for an AI win and 0 for an AI loss, and at each turn, choose the available move with the highest total expected branch reward (figure 3*a*). By contrast, MCTS OSAS algorithms use a graded reward function that returns a range of reward values depending on how close the final game state is to a target game state (figure 3*b*), which can be set at liberty. In *Explorers* *vs* *Owls*, players can win or lose to different degrees, expressed in the final score difference between them and the AI opponent. Thus, our MCTS OSAS target game state took the form of a *target PWM*. For our three conditions, we set the balanced condition target PWM to 0 to aim for a draw. The easy and hard conditions had target PWM of 15 and −15 respectively. Pilot testing showed that these targets were experienced as markedly different, the median player score during pilots was 30 points, so a win margin of 15 often means that one player scored 50% points more than the other. (The theoretically possible PWM range spans 162 points in our game, from −81 to 81). We used the ‘True POSAS’ [44] variant of MCTS OSAS which is designed for turn-based games where the human player moves first and so has an advantage over the AI opponent. True POSAS reduces this first move advantage by aiming for a slightly higher target score than specified, which is done by adding the maximum score per move (3 points) to the target PWM to create a ‘flattened cone’ reward function shown in figure 3*b*.

We ran an informal pilot study (*n* = 29) to ensure the game was easy to understand, fun to play, featured strategic depth and to determine which differences in PWM players experienced as markedly different.

### 2.1. Implementation and performance

The game was written using the Unity game engine in C# and compiled to run as a web page using WEBGL/WASM technologies. Although faster than JavaScript, this is still considerably slower than compiling as a native desktop application. As with MCTS algorithms more generally, the MCTS OSAS AI opponent performs stronger—and thus, achieves target outcomes more accurately—if it has more computing cycles to decide on its move. The more complete playthroughs of the game (known as iterations) it can simulate, the more likely it is to meet its target win margin. However, if the system takes too long to make its move, this would make the game less engaging [50] and could thus confound results. In our online experiment, participants used their own desktop computers with a wide range of performance. During game development and pilot testing, we found that 7500 iterations sufficed to give a strong game while taking an acceptable waiting time (mean of less than 2.5 s) on a current mid-range computer. We performed two checks during the experiment to ensure that all participants experienced similar opponent move times and that variation in AI speed and strength did not confound results. The first check, at the very start of the experiment, was to test the speed of the computer by measuring the time it took to perform a computationally demanding task (creating 100 000 memory objects). Those participants whose computers took too long (greater than 0.17 s) were asked to withdraw from the experiment and not able to progress any further. The second check was to record the maximum time for any AI move during the whole experiment. If this was greater than 6 s, then that participant's data was excluded from the experiment. Note that the algorithm takes longest to make the first few moves and then speeds up considerably; a maximum move time of 6 s (on the first move) would mean that the average move time during a game ends up at around 2.5 s. Visually animating the move the player inputted takes 1–2 s, during which time the AI already processed its response. Thus, with a total average 2.5 s move time, the average wait time perceived by the player was less than 1 s.

### 2.2. Participants

We recruited participants using the Prolific online recruitment platform. The experiment was completed by 368 participants. In accordance with our pre-registered criteria, we rejected 51 whose computers took more than 6 s to make a move, three who failed the attention check and three who took more than 20 min to perform the experiment. This left 311 participants, 100 in the easy condition, 109 in the balanced condition and 102 in the hard condition. Ages ranged from 18 to 56 (mean = 30.4). Of these 158 were female, 151 were male, one non-binary and one preferred not to give a gender. Participants were allocated to a condition in turn, so the first participant was the easy condition, the second participant the balanced condition and so on.

### 2.3. Measures

We measured experiential enjoyment using the intrinsic motivation inventory (IMI) enjoyment subscale [51], which is the most commonly used [52] validated measure of experiential enjoyment. We measured behavioural engagement as the percentage of players in each condition that voluntarily chose to play the game again after they were told that mandated experimental play was over, emulating common voluntary time on task measures [53]. We measured agency using an established single-item agency scale [54]. Consent, demographic information and all self-report measures were recorded using online forms integrated into the game. As described, difficulty-skill ratios were measured as PWMs (PWM) = *player score – AI score*. Following a reviewer suggestion, we added a measurement for whether players improved at the game (PWM improvement), calculated as the difference in PWM between the last and first full game = *PWM*_last game_ – *PWM*_first game_.

### 2.4. Procedure

The experiment starts by checking the speed of participants' computers. Those with slow computers are asked to withdraw from the experiment and prevented from progressing. The remaining participants are blindly assigned to one of the three conditions and complete an informed consent procedure and then answer some demographic questions. They then complete a short interactive tutorial to teach them how to play the game. This consists of a game of *Explorers vs Owls* played on a 4 × 4 board with one piece per player. For the tutorial, the AI opponent always aims to draw regardless of the condition. Once the tutorial is finished, participants play two games of *Explorers* *vs* *Owls* on a 7 × 7 board, with each player having three figures. For both of these games, the AI opponent aims for the PWM specified by the condition that the player is in. After playing the two games, they complete a single-question agency assessment followed by the seven-question enjoyment subscale of the IMI questionnaire. They are then asked if they noticed any bugs or technical issues. Finally, they are told that they have completed enough games to receive their payment and asked if they want to play another game or finish the experiment. Choosing either option brings up the Prolific study completion page in another browser tab, but choosing to play again also allows them to play the main game again.

## 3. Results

### 3.1. Validity of approach

**Figure 4.**
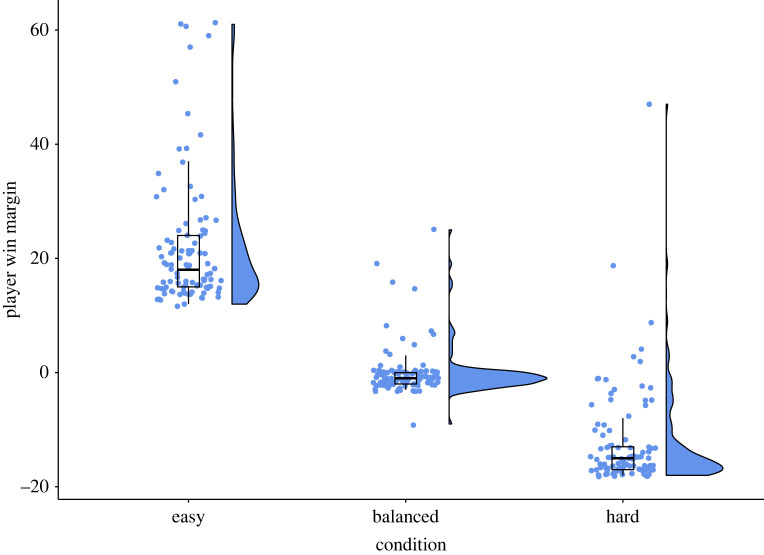
PWM (player score minus AI score) for the last match played in each condition. In the balanced (target PWM = 0) and hard (target PWM = −15) conditions, the majority of matches achieved their target. In the easy condition (target PWM = 15), most matches hit the target of 15, but players were more likely to exceed the target than in the other conditions. Nevertheless, our system successfully manipulated difficulty-skill ratios on an individual player level.

Since our study depended on effectively manipulating individual difficulty-skill ratios in the form of PWM, we first tested whether this manipulation succeeded. A Kruskal–Wallis test showed a significant difference in PWM between conditions with an extremely large effect size (H_2_ = 244, *p* < 0.001, *ε*^2^ = 0.786). Pairwise comparisons showed a significant difference (*p* < 0.001) between all conditions, with the easy condition the highest and the hard condition the lowest. The median PWM for each condition was very close to the target aimed for, and players did not meaningfully improve their PWM over games, with a median improvement of 1 (out of 162) points for easy, 0 for draw, and −1 for hard (table 1, figure 4). This supports H3 and H3a. To determine whether players felt they were improving at the game, we calculated the difference in PWM between the last and first game (PWM improvement). For all conditions, this was very low with a median absolute value of 1 or less.

**Table 1.** In each of three the conditions, the AI opponent aims for a different target win margin to give a different difficulty-skill ratio. The outcomes show that the AI opponent succeeded in that, both per game and across games: the differences in PWMs between a player's first and last games (PWM improvement) hovered close to zero. Meanwhile, participant agency, experienced enjoyment and behavioural engagement do not show significant differences between conditions.

condition	easy	balanced	hard
*N*	100	109	102
target PWM^a^	15	0	−15
**actual game outcomes**			
actual PWM mean (s.d.)^a^	22 (11.5)	−0.06 (4.32)	−12.4 (8.88)
actual PWM Median^a^	18	−1	−15
player wins %^b^	100	12.8	5.9
player draws %^b^	0	23.9	0
player loses %^b^	0	63.3	94.1
PWM improvement mean (s.d.)	2.59 (10.94)	0.064 (5.57)	1.33 (10.22)
PWM improvement median^a^	1	0	−1
**participant experience and behaviour**			
agency mean (s.d.)	78.7 (18.9)	72.8 (22.7)	71.9 (25.1)
experiential enjoyment (IMI) mean (s.d.)	39.3 (6.93)	38.2 (7.98)	37.5 (9.21)
behavioural engagement (% of players that chose to play again)^b^	20.0	29.0	30.0

^a^Final game scores (player score minus AI score), where positive values equal player wins and negative player losses; 0 equals a draw.

^b^Categorical values which do not have an associated s.d.

Next, we checked whether players might have noticed our manipulation, resulting in reduced perceived agency over the game outcome. A Kruskal–Wallis test found no significant difference in perceived agency between conditions (H_2_ = 2.94, *p* = 0.230, *ε*^2^ = 0.010), suggesting that players did not notice our manipulation, or if they did, not in a way that differentially affected our conditions (table 1).

### 3.2. Main effects

**Figure 5.**
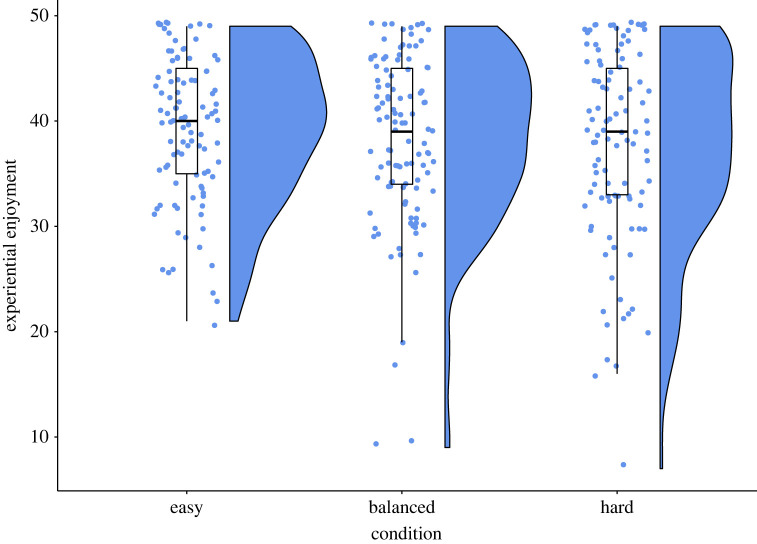
Experiential enjoyment for each condition as measured by the IMI enjoyment questionnaire. While more participants reported low enjoyment in the balanced and hard conditions than in the easy condition, we see no statistically significant differences in enjoyment between different difficulty-skill ratios.

Counter to our hypothesis (H1), an ANOVA found that experiential enjoyment did not differ significantly between the three conditions; *F*_2,308_ = 1.29, *p* = 0.277, $\;\eta_{p}^{2}=0.008$ (table 1, figure 5). A Shapiro–Wilk test indicated that the enjoyment scores may not be normally distributed (*p* < 0.001), so we performed a Kruskal–Wallis test, which also did not find a significant difference; H_2_ = 1.25, *p* = 0.534, *ε*^2^ = 0.04. Similarly, counter to H2, we found no significant difference in behavioural engagement between conditions, as determined by a Chi-square test of independence between conditions in the share of participants who wished to play the game again: $\chi_{2,311}^{2}=3.40$, *p* = 0.183 (table 1). As pre-registered, since H1 and H2 were not supported, we did not conduct *post hoc* tests whether the balanced condition featured the highest enjoyment and engagement (H1a, H2a).

We performed exploratory analyses to understand the relationships between enjoyment, engagement and player score. Binomial logistic regressions found a significant correlation (Z = 4.76, *p* < 0.001, McFadden's *r*^2^ = 0.078) between engagement and enjoyment, and another significant correlation (although with a smaller effect size) between engagement and whether players won or not (Z = 2.10, *p* < 0.036, McFadden's *r*^2^ = 0.011). However, there was no significant correlation between enjoyment and whether players won, lost or drew (*Z* = −1.63, *p* = 0.104, McFadden's *r*^2^ = 0.007).^1^ A further exploratory analysis suggested by a reviewer found no significant correlations between improvements from first game to last game (PWM improvement) and enjoyment (*r* = 0.039, *p* = 0.497) or engagement (*Z* = −0.116, *p* = 0.908, McFadden's *r*^2^ = 0.007).

## 4. Discussion

Prior work on whether task engagement and enjoyment are optimized when difficulty and skill are in balance has suffered from lacking theoretical formalization of ‘balance’ as well as questionable measurement practices. To avoid these issues and advance available methods, we developed a skill-based online game that uses an MCTS OSAS algorithm to control individual-level difficulty-skill ratios. Our system was highly effective at affording the target skill-difficulty ratio, expressed in PWMs: the achieved median PWMs deviated 0 (hard condition), 1 (balanced condition) and, at most, 3 (easy condition) points from the target PWMs on a scale that spans 162 points (−81 to 81). Hundred per cent of players in the easy condition won, 94.1% of players in the hard condition lost and 23.9% in the balanced condition got a draw. Similarly, players did not noticeably improve between games, with the median PWM improvement from first to last game being 1, 0 and −1 for the easy, balanced and hard conditions, respectively. This suggests that the AI adapted as desired to any actual player learning between games, thereby eliminating a (sense of) progress or improvement as a potential confound. These are excellent results, especially given that our system targeted a PWM *corridor* for each condition, which spanned narrow wins and losses for the balanced condition (see Materials and methods).

As *Explorers* *vs* *Owls* is a zero-sum game, there are theoretical limits on the AI opponent's performance. When the AI is aiming to lose and the human player consistently makes the worst possible moves, a drawn game results. Similarly, when the AI is aiming to win and the player consistently makes optimal moves, this will also result in a draw. In our study, there was no evidence of these boundary conditions ever being reached. However, in the hard and easy conditions, deviations drifted toward greater player wins. We believe this is due to the deep forward-planning horizon *Explorers* *vs* *Owls* affords, together with its encircling mechanic and small board: players experienced in similar games (e.g. *Go*) would be able to circle AI opponent figures in or circle off a large point-scoring territory for their own figures with early moves that would lead to larger PWMs in the late game. Given enough computing cycles, MCTS OSAS should be principally able to ‘foresee’ such moves and achieve PWMs even closer to the target. Our participants' hardware and acceptable wait time put a pragmatic limit on available computing cycles. Future server-side implementations of the AI system could easily overcome this issue.

A second concern with our method is that participants might have noticed our difficulty manipulation, undermining their sense of agency, which could confound results. However, participants in all conditions reported high levels of agency over their gameplay, with no significant differences between conditions.

In sum, our test game suite and general approach of using AI-controlled opponents show promise as a scalable, unobtrusive method for manipulating and studying objective difficulty-skill ratios. While MCTS algorithms have proven particularly apt for tactical, turn-based games like our test game, there are already working implementations of MCTS OSAS for real-time action games [44]. Extending and validating our approach for such and other game genres is one avenue for future work, also to establish the generalizability of our findings. Another present limitation is that our system can control and capture difficulty-skill ratios, but not independent absolute player skill or opponent difficulty measures. We have already demonstrated that such measures can be derived from MCTS OSAS play data using Elo-like approaches [43], but implementing and validating this for experimental research remains future work.

Contrary to hypotheses derived from flow and intrinsic motivation theories, we found no significant difference in enjoyment or engagement between conditions with markedly different objective difficulty-skill ratios, and with that, also no inverted-U pattern apexing at some difficulty-skill balance. Our results also contradict two prior large-scale online studies [20,38] that found linear rather than inverted-U relations between objective difficulty-skill ratio and behavioural engagement; that said, exploratory analyses found a (small) correlation between behavioural engagement (choosing to play again) and player wins, which broadly fits a linear relation where easier games equal more engagement.

How do we explain these different results? As the replication crisis has shown, even effects considered well-supported by dozens of experimental studies can fail to replicate or shrink in effect size when put to rigorous replication tests [55]. Of course, no single study, even if pre-registered and adequately powered, can invalidate a whole body of work. But our results fit with the small relation between balance and intrinsic motivation found in previous meta-analysis (*z* = 0.24) [23], and other prior work finding that difficulty-skill balance explains little of the overall observed variance in subjective experience [22]. Assuming our null finding is true, previous results supporting an inverted-U relation may have been artefacts of small sample sizes, unconscious or conscious uses of researcher degrees of freedom, tautological independent and dependent self-report measures (which make up the majority of current work [23]), or an inability to afford targeted skill-difficulty ratios.

A second explanation is unexplored moderators or boundary conditions. Studies observing an inverted-U [37], linear [20,38] or no (e.g. our study) relation may each have found true effects that, however, do not generalize beyond their particular population, context or task.

Our study did not include a control condition without any DDA. The consistently high reported enjoyment and fact that players did not notice the DDA manipulation could thus be attributed to DDA use *as such*, regardless of targeted outcome. What ‘matters’ in the long run (over extended play) is difficulty that follows the player's learning curve. This is possible, but outside the scope of our study, as players only played two full matches in sequence, probably not allowing for such sequential effects to show (or moderate results). Future studies could investigate the effects of removing DDA by fixing it on a target play strength rather than outcome over extended matches, which raises the interesting open question of what play strength(s) would be ecologically valid operationalizations of default difficulty.

Another possibility is that all three DDA conditions were within the ‘optimal zone’ and so increased enjoyment or engagement equally—i.e. the difficult and easy manipulations were too subtle. Against that stand our pilot studies showing that players did notice the differences in play strength, and that 94.1% of players lost in the hard condition and all won in the easy condition; so it seems unlikely that these should have *no* impact on experience and behaviour, if their relation with difficulty-skill balance indeed forms a graded curve, not a step function. Still, more extreme conditions could be tested.

An additional proposed moderator is awareness and deliberate choice of a particular task difficulty, which one study found to turn a linear into an inverted-U relation [20]. Relatedly, DDA has also been found to be more effective if players are unaware of it [35,36], though there is also evidence that awareness of DDA could have the opposite effect [49]. Either way, the consistent and consistently high levels of reported player agency across conditions suggest that differences in awareness between conditions did not moderate our results.

A final related possible moderator is gaming identity and values: particularly male ‘gamer’ communities put value on ‘real’ games being difficult and thus, meritocratic tests of skill [56,57]. This valorization is not necessarily shared by the wider population from which we drew our participants, as is demonstrated by the broad popularity of so-called casual games [58]. In previous studies, self-selecting ‘gamer’ (or sports athlete) populations might self-report to prefer what they believe to be ‘balanced’ or even ‘hard’ challenges as a result of their particular values and taste, and/or to signal and affirm their gamer (or athlete) identity.

Third is the question of construct validity of our study. While we tested the general relation between objective difficulty-skill ratios and enjoyment and engagement proposed by several theories, the arguably most directly relevant theory is flow theory, which consistently emphasizes that difficulty-skill balance is a *subjective perception*: ‘*subjective challenges and subjective skills, not objective ones, [...] influence the quality of a person's experience*’ [31]. However, Csikszentmihalyi later posited a similar relation for *objective* difficulty-skill balance and flow, and presented as evidence objective difficulty-skill ratios as support for flow theory [37]. It indeed seems highly implausible that subjective and objective difficulty-skill ratios should be completely disjunct, especially under frequent extended performance feedback as found in gameplay, which would inform and correct subjective balance assessment and expectations. If they were strongly disjunct, this would be important if bad news for DDA and intelligent tutoring systems. A more differentiated construct validity critique of our study is that later versions of flow theory propose that flow (and with it, enjoyment) only manifest under balanced *high* skill and *high* difficulty [31], which we did not ensure. Again, other intrinsic motivation theories [6] subscribing to the inverted-U model do not set out this necessary condition, and Csikszentmihalyi himself co-conducted studies that expressly forfeited controlling for high skill and difficulty [37]. But we concur that future replications of our study could do so.

Fourth, we see two possible limitations of the present study that could have produced a false negative finding. The first is that our game may have been paradoxically too enjoyable for reasons other than difficulty-skill balance. Current gaming motivation and experience models generally recognize multiple sources of enjoyment and engagement beyond flow or competence need satisfaction [59,60], which are usually causally linked to difficulty-skill balance [23]. Specifically, there is emerging evidence and argument that curiosity, stoked by e.g. uncertain or novel experiences, could explain variance in enjoyment and engagement typically ascribed to competence or flow [20]. Now we intentionally designed *Explorers* *vs* *Owls* to be as ecologically valid and fun as possible; the game offers gameplay not commonly found in other casual online games, and gameplay was comparatively short (mean time around 7 min). Thus, the game's novelty may have been so strong as to produce ceiling effects in reported enjoyment and engagement, overshadowing any additional real differences produced by our difficulty-skill ratio manipulation, even though we used a pilot study to ensure our manipulation was clearly noticeable. This is particularly possible given our Prolific sample probably included many professional online study participants who were used to less engaging experimental tasks. While this explanation is worthy of follow-on studies, if true, it would also suggest that difficulty-skill balance is a less relevant factor for task experience and motivation than previously thought.

The second possible confound of our online sample is that participants were paid to play our game and may have participated in it as a form of (low-waged) work. This may have produced careless responding (which we tried to control for with attention checks), but also different responses to difficulty, with participants satisficing their way through the perceived-mandatory game task with minimum effort and involvement. That does not quite fit the high reported enjoyment and approximately 26% of participants across conditions choosing to play again even after they were told the paid task was over, but it remains an important general validity threat of paid studies on gameplay. Future studies could seek to replicate our findings with a more naturistic play environment.

Given that we found that difficulty-skill ratios (operationalized as game outcomes) on their own do not impact enjoyment or engagement, what factors are likely to create these positive experiences in gameplay and other tasks with ‘balanced’ challenge? One possible explanation already mentioned is other motivational mechanisms not directly tied to challenge, such as curiosity [20]: directly ‘balancing’ task difficulty against growing player skill indirectly produces diverse and novel challenges. Another explanation is that people are intrinsically motivated by and experience enjoyment not over absolute success, failure or challenge, but relative improvement or progress over time—a sense of learning progress [61,62]. Recent theoretical work suggests that intrinsic motivation models focusing on improvement (like empowerment maximization or uncertainty reduction) offer more coherent accounts of the appeal of balanced challenge than currently dominant models like flow or self-determination theory [63]. While our exploratory analyses found no correlations between PWM improvement and enjoyment or engagement, our AI successfully adapted to any possible player skill improvement, as seen in the near-zero objective PWM improvements between first and last game. Thus, our manipulation successfully *suppressed* potential objective and perceived improvement, and with that, could have obscured possible true effects. Future work could test this improvement hypothesis by using our system to artificially induce different rates of improvement.

## 5. Conclusion

The idea that the balance of difficulty and skill in a task affects our enjoyment and engagement holds intuitive appeal. By way of flow theory and other theories of intrinsic motivation, it has deeply influenced how we think about and organize many domains of our life, such as education, sports, work and entertainment. Yet despite decades of research, rigorous studies probing whether and where an objective ‘sweet spot’ of balance exists have been sparse and hard to conduct. Our study not only suggests that difficulty-skill balance may not affect engagement and enjoyment as much or directly as previously thought. It also reinforces evidence that digital games provide a controlled and ecologically valid environment to investigate social science questions like skill-difficulty balance. Contemporary game AI techniques like MCTS OSAS allow us to unobtrusively control and vary participants' individual experimental experience within those environments with a rigour and scale unavailable with previous techniques.

## Data Availability

All experimental data and questionnaire items and the code used to run the game are available in an OSF repository at https://osf.io/8wtve/ (folder ‘Experiment 1’).

## Footnotes

1 We erroneously pre-registered that we would perform a Pearson correlation between engagement and enjoyment, which is not possible, since engagement is a categorical variable (play again or not). This should have been a binomial logistic regression which is reported here.

## References

[1] Teigen KH. 1994 Yerkes-Dodson: a law for all seasons. Theory Psychol. **4**, 525-547. (10.1177/0959354394044004)

[2] Berlyne DE. 1970 Novelty, complexity, and hedonic value. Percept. Psychophys. **8**, 279-286. (10.3758/BF03212593)

[3] Chmiel A, Schubert E. 2017 Back to the inverted-U for music preference: a review of the literature. Psychol. Music **45**, 886-909. (10.1177/0305735617697507)

[4] Veldhuizen MG, Van Rooden AP, Kroeze JH. 2006 Dissociating pleasantness and intensity with quinine sulfate/sucrose mixtures in taste. Chem. Senses **31**, 649-653. (10.1093/chemse/bjl005) 16793856

[5] Csikszentmihalyi M. 1991 Flow: the psychology of optimal experience, vol. 41. New York, NY: HarperPerennial.

[6] Deci EL, Ryan RM. 1984 Conceptualizations of intrinsic motivation and self-determination. In Intrinsic motivation and self-determination in human behavior, pp. 11-40. Berlin, Germany: Springer.

[7] Perttula A, Kiili K, Lindstedt A, Tuomi P. 2017 Flow experience in game based learning – a systematic literature review. Int. J. Ser. Games **4**, 57-72. (10.17083/ijsg.v4i1.151)

[8] Nielsen K, Cleal B. 2010 Predicting flow at work: investigating the activities and job characteristics that predict flow states at work. J. Occup. Health Psychol. **15**, 180-190. (10.1037/a0018893) 20364915

[9] Tims M, Bakker AB. 2010 Job crafting: towards a new model of individual job redesign. SA J. Industrial Psychol. **36**, 1-9. (10.4102/sajip.v36i2.841)

[10] Chan TS, Ahern TC. 1999 Targeting motivation—adapting flow theory to instructional design. J. Educ. Comput. Res. **21**, 151-163. (10.2190/UJ04-T5YB-YFXE-0BG2)

[11] Jackson SA, Csikszentmihalyi M. 1999 Flow in sports. Champaign, IL: Human Kinetics.

[12] Swann C, Keegan RJ, Piggott D, Crust L. 2012 A systematic review of the experience, occurrence, and controllability of flow states in elite sport. Psychol. Sport Exercise **13**, 807-819. (10.1016/j.psychsport.2012.05.006)

[13] Sweetser P, Wyeth P. 2005 GameFlow: a model for evaluating player enjoyment in games. Comput. Entertain. (CIE) **3**, 3. (10.1145/1077246.1077253)

[14] Chen J. 2007 Flow in games (and everything else). Commun. ACM **50**, 31-34. (10.1145/1232743.1232769)

[15] Mousavinasab E, Zarifsanaiey N, R. Niakan Kalhori S, Rakhshan M, Keikha L, Ghazi Saeedi M. 2021 Intelligent tutoring systems: a systematic review of characteristics, applications, and evaluation methods. Interact. Learn. Environ. **29**, 142-163. (10.1080/10494820.2018.1558257)

[16] Guo B, Liu Y, Wang L, Li VOK, Lam JCK, Yu Z. 2018 Task allocation in spatial crowdsourcing: current state and future directions. IEEE Internet Things J. **5**, 1749-1764. (10.1109/JIOT.2018.2815982)

[17] Sarkar A, Williams M, Deterding S, Cooper S. 2017 Engagement effects of player rating system-based matchmaking for level ordering in human computation games. In Proc. of the 12th Int. Conf. on the Foundations of Digital Games, Hyannis, MA, August, pp. 1-10. New York, NY: Association for Computing Machinery.

[18] Graepel T, Herbrich R. 2006 Ranking and matchmaking. Game Dev. Magazine **25**, 34-54.

[19] Zohaib M. 2018 Dynamic difficulty adjustment (DDA) in computer games: a review. Adv. Human-Comput. Interact. **2018**, 5681652. (10.1155/2018/5681652)

[20] Lomas JD, Koedinger K, Patel N, Shodhan S, Poonwala N, Forlizzi JL. 2017 Is difficulty overrated? The effects of choice, novelty and suspense on intrinsic motivation in educational games. in Proc. of the 2017 CHI Conf. on Human Factors in Computing Systems. New York, NY: Association for Computing Machinery.

[21] Ellis GD, Voelkl JE, Morris C. 1994 Measurement and analysis issues with explanation of variance in daily experience using the flow model. J. Leisure Res. **26**, 337-356. (10.1080/00222216.1994.11969966)

[22] Løvoll HS, Vittersø J. 2014 Can balance be boring? A critique of the ‘challenges should match skills' hypotheses in flow theory. Social Indicators Res. **115**, 117-136. (10.1007/s11205-012-0211-9)

[23] Fong CJ, Zaleski DJ, Leach JK. 2015 The challenge–skill balance and antecedents of flow: a meta-analytic investigation. J. Positive Psychol. **10**, 425-446. (10.1080/17439760.2014.967799)

[24] Elson M. 2019 Examining psychological science through systematic meta-method analysis: a call for research. Adv. Methods Practices Psychol. Sci. **2**, 350-363. (10.1177/2515245919863296)

[25] Parry DA, Davidson BI, Sewall CJ, Fisher JT, Mieczkowski H, Quintana DS. 2021 A systematic review and meta-analysis of discrepancies between logged and self-reported digital media use. Nat. Hum. Behav. **5**, 1535-1547. (10.1038/s41562-021-01117-5) 34002052

[26] Flake JK, Fried EI. 2020 Measurement schmeasurement: questionable measurement practices and how to avoid them. Adv. Methods Practices Psychol. Sci. **3**, 456-465. (10.1177/2515245920952393)

[27] Szollosi A, Donkin C. 2021 Arrested theory development: the misguided distinction between exploratory and confirmatory research. Perspect. Psychol. Sci. **16**, 717-724. (10.1177/1745691620966796) 33593151

[28] Oberauer K, Lewandowsky S. 2019 Addressing the theory crisis in psychology. Psychon. Bull. Rev. **26**, 1596-1618. (10.3758/s13423-019-01645-2) 31515732

[29] Csikszentmihalyi M, Nakamura J. 2010 Effortless attention in everyday life: a systematic phenomenology. In Effortless attention: a new perspective in the cognitive science of attention and action (ed. B Bruya), pp. 179-189. Cambridge, MA: MIT Press.

[30] Keller J, Bless H. 2008 Flow and regulatory compatibility: an experimental approach to the flow model of intrinsic motivation. Pers. Soc. Psychol. Bull. **34**, 196-209. (10.1177/0146167207310026) 18212330

[31] Nakamura J, Csikszentmihalyi M. 2014 The concept of flow. In Flow and the foundations of positive psychology, pp. 239-263. Berlin, Germany: Springer.

[32] Klarkowski M, Johnson D, Wyeth P, McEwan M, Phillips C, Smith S. 2016 Operationalising and evaluating sub-optimal and optimal play experiences through challenge-skill manipulation. In Proc. of the 2016 CHI Conf. on Human Factors in Computing Systems, San Jose, CA, 7–12 May, pp. 5583-5594. New York, NY: Association for Computing Machinery.

[33] Ulrich M, Keller J, Hoenig K, Waller C, Grön G. 2014 Neural correlates of experimentally induced flow experiences. Neuroimage **86**, 194-202. (10.1016/j.neuroimage.2013.08.019) 23959200

[34] Keller J, Bless H, Blomann F, Kleinböhl D. 2011 Physiological aspects of flow experiences: skills-demand-compatibility effects on heart rate variability and salivary cortisol. J. Exp. Soc. Psychol. **47**, 849-852. (10.1016/j.jesp.2011.02.004)

[35] Hunicke R. 2005 The case for dynamic difficulty adjustment in games. In Proc. of the 2005 ACM SIGCHI Int. Conf. on Advances in Computer Entertainment Technology, Valencia, Spain, 15–17 June, pp. 429-433. New York, NY: Association for Computing Machinery.

[36] Abuhamdeh S, Csikszentmihalyi M. 2012 The importance of challenge for the enjoyment of intrinsically motivated, goal-directed activities. Pers. Soc. Psychol. Bull. **38**, 317-330. (10.1177/0146167211427147) 22067510

[37] Abuhamdeh S, Csikszentmihalyi M, Jalal B. 2015 Enjoying the possibility of defeat: outcome uncertainty, suspense, and intrinsic motivation. Motivation Emot. **39**, 1-10. (10.1007/s11031-014-9425-2)

[38] Lomas D, Patel K, Forlizzi JL, Koedinger KR. 2013 Optimizing challenge in an educational game using large-scale design experiments. In Proc. of the SIGCHI Conf. on Human Factors in Computing Systems, Paris, France, 27 April–2 May, pp. 89-98. New York, NY: Association for Computing Machinery.

[39] Ronimus M, Kujala J, Tolvanen A, Lyytinen H. 2014 Children's engagement during digital game-based learning of reading: the effects of time, rewards, and challenge. Comput. Educ. **71**, 237-246. (10.1016/j.compedu.2013.10.008)

[40] Gray W. 2017 Game-XP: action games as experimental paradigms for cognitive science. Top. Cogn. Sci. **9**, 289-307. (10.1111/tops.12260) 28296290

[41] Elo A. 1978 The rating of chess players, past and present. New York, NY: Arco.

[42] Herbrich R, Minka T, Graepel T. 2006 Trueskill™: a Bayesian skill rating system. In Proc. of the 19th Int. Conf. on Neural Information Processing Systems, Vancouver, Canada, December, pp. 569-576. Cambridge, MA: MIT Press.

[43] Demediuk S, Tamassia M, Raffe WL, Zambetta F, Mueller FF, Li X. 2018 Measuring player skill using dynamic difficulty adjustment. In Proc. of the Australasian Computer Science Week Multiconference, Queensland, Australia, 29 January–2 February, pp. 1-7. New York, NY: Association for Computing Machinery.

[44] Demediuk S, Tamassia M, Raffe WL, Zambetta F, Li X, Mueller F. 2017 Monte Carlo tree search based algorithms for dynamic difficulty adjustment. In 2017 IEEE Conf. on Computational Intelligence and Games (CIG). IEEE.

[45] Browne CB et al. 2012 A survey of Monte Carlo tree search methods. IEEE Trans. Comput. Intelligence AI games **4**, 1-43. (10.1109/TCIAIG.2012.2186810)

[46] Edelkamp S, Gath M, Greulich C, Humann M, Herzog O, Lawo M. 2016 Monte-Carlo tree search for logistics. In Commercial transport (eds U Clausen, H Friedrich, C Thaller, C Geiger), pp. 427-440. Berlin, Germany: Springer.

[47] Perez D, Rohlfshagen P, Lucas SM. 2012 Monte-Carlo tree search for the physical travelling salesman problem. In European Conf. on the Applications of Evolutionary Computation. Berlin, Germany: Springer.

[48] Silver D et al. 2016, Mastering the game of Go with deep neural networks and tree search. Nature **529**, 484-489. (10.1038/nature16961) 26819042

[49] Denisova A, Cairns P. 2019 Player experience and deceptive expectations of difficulty adaptation in digital games. Entertain. Comput. **29**, 56-68. (10.1016/j.entcom.2018.12.001)

[50] Nah FFH. 2004 A study on tolerable waiting time: how long are web users willing to wait? Behav. Inform. Technol. **23**, 153-163. (10.1080/01449290410001669914)

[51] Ryan RM. 1982 Control and information in the intrapersonal sphere: an extension of cognitive evaluation theory. J. Pers. Soc. Psychol. **43**, 450. (10.1037/0022-3514.43.3.450)

[52] Mekler ED, Bopp JA, Tuch AN, Opwis K. 2014 A systematic review of quantitative studies on the enjoyment of digital entertainment games. In Proc. of the 32nd Annual ACM Conf. on Human Factors in Computing Systems, Ontario, Canada, 26 April–1 May, pp. 927-936. New York, NY: Association for Computing Machinery.

[53] Birk MV, Atkins C, Bowey JT, Mandryk RL. 2016 Fostering intrinsic motivation through avatar identification in digital games. In Proc. of the 2016 CHI Conf. on Human Factors in Computing Systems, San Jose, CA, 7–12 May, pp. 2982-2995. New York, NY: Association for Computing Machinery.

[54] Pailhès A, Kumari S, Kuhn G. 2020 The magician's choice: providing illusory choice and sense of agency with the equivoque forcing technique. J. Exp. Psychol. **150**, 1358-1372. (10.1037/xge0000929) 33252983

[55] Shrout PE, Rodgers JL. 2018 Psychology, science, and knowledge construction: broadening perspectives from the replication crisis. Annu. Rev. Psychol. **69**, 487-510. (10.1146/annurev-psych-122216-011845) 29300688

[56] Paul CA. 2018 The toxic meritocracy of video games: why gaming culture is the worst. Minneapolis, MN: University of Minnesota Press.

[57] Consalvo M, Paul CA. 2013 Welcome to the discourse of the real: constituting the boundaries of games and players. In 8th Int. Conf. on the Foundations of Digital Games, Chania, Greece, 14–17 May, pp. 1-8. Santa Cruz, CA: SASDG.

[58] Kultima A. 2009 Casual game design values. In Proc. of the 13th Int. MindTrek Conf.: Everyday Life in the Ubiquitous Era, Tampere, Finland, 30 September–2 October, pp. 58-65. New York, NY: Association for Computing Machinery.

[59] Abeele VV, Spiel K, Nacke L, Johnson D, Gerling K. 2020 Development and validation of the player experience inventory: a scale to measure player experiences at the level of functional and psychosocial consequences. Int. J. Hum. Comput. Stud. **135**, 102370. (10.1016/j.ijhcs.2019.102370)

[60] Boyle EA, Connolly TM, Hainey T, Boyle JM. 2012 Engagement in digital entertainment games: a systematic review. Comput. Hum. Behav. **28**, 771-780. (10.1016/j.chb.2011.11.020)

[61] Oudeyer PY, Kaplan F. 2009 What is intrinsic motivation? A typology of computational approaches. Front. Neurorob. **1**, 6. 10.3389/neuro.12.006.2007 PMC2533589 18958277

[62] Gottlieb J, Oudeyer PY. 2018 Towards a neuroscience of active sampling and curiosity. Nat. Rev. Neurosci. **19**, 758-770. (10.1038/s41583-018-0078-0) 30397322

[63] Deterding S, Andersen MM, Kiverstein J, Miller M. 2022 Mastering uncertainty: a predictive processing account of enjoying uncertain success in video game play. Front. Psychol. **13**, 4214. (10.3389/fpsyg.2022.924953) PMC9363017 35959012

